# A study on the double-edged sword effect of inclusive leadership on employees’ work behaviour—dual path perspective of cognition and affection

**DOI:** 10.3389/fpsyg.2024.1310204

**Published:** 2024-05-07

**Authors:** Hao Chen, Jiaying Bao, Liang Wang, Zihan Zhang

**Affiliations:** ^1^School of Public Health and Management, Youjiang Medical University for Nationalities, Baise, Guangxi, China; ^2^School of Languages and Cultures, Youjiang Medical University for Nationalities, Baise, Guangxi, China; ^3^School of Economics and Management, Wuxi Vocational Institute of Arts and Technology, Wuxi, Jiangsu, China; ^4^School of Economics and Management, Wuhan University, Wuhan, Hubei, China

**Keywords:** inclusive leadership, psychological entitlement, state gratitude, work withdrawal behaviour, proactive behaviour, narcissistic personality

## Abstract

In order to cope with the volatile social environment and organisational change, more and more scholars call on leaders to stimulate subordinate effectiveness to a greater extent with inclusive behaviour. Existing studies focus on the positive impact of inclusive leadership, but ignore its potential negative impact. This study integrates Cognition-affection Personality System Theory to explore the double-edged sword mechanism of inclusive leadership on subordinates’ work behaviour. Through the data analysis of 518 paired questionnaires collected in three stages, the results are as follows: Inclusive leadership has a positive impact on subordinates’ psychological entitlement and state gratitude; Psychological entitlement and state gratitude play mediation roles not only between inclusive leadership and work withdrawal behaviour, but also between inclusive leadership and active behaviour; Subordinate narcissistic personality moderates the positive effect of inclusive leadership on psychological entitlement and state gratitude, and then moderates the indirect effect of inclusive leadership on subordinate work withdrawal behaviour and proactive behaviour through psychological entitlement and state gratitude. The above results expand the research on the action mechanism and boundary conditions of inclusive leadership in Chinese organisational context, and provide practical guidance for organisational managers to effectively conduct inclusive leadership.

## Introduction

With the rapid evolution of the organisational structure, the diverse, individuality, differentiated and autonomous needs of employees are increasing day by day, and in order to effectively cope with the increasingly complex and uncontrollable management environment and to lead the organisation to develop steadily in the turbulent external environment, an inclusive leadership behaviour, which carries the Chinese culture of “Inclusive and having tolerance,” has come into being. Inclusive leadership, with “inclusiveness” at its core (Chen and Cheng, 2021), is a leadership style that integrates respect, support, tolerance, appreciation and motivation for subordinates (AlMulhim and Mohammed, 2023). Numerous studies have confirmed that inclusive leadership has a positive effect on subordinates’ attitudes, behaviour and performance (Javed et al., 2019; Song et al., 2023), which can both effectively improve subordinates’ work efficiency (Sürücü et al., 2023) and be able to build harmonious interactions with subordinates (Yasin et al., 2023).

Although existing research generally affirms the effectiveness and positive effect of inclusive leadership (Morinaga et al., 2023), some scholars have questioned and challenged this mainstream hypothesis, arguing that the high level of affinity, security and inclusiveness exhibited by inclusive leadership over time can have negative mechanisms of action on subordinates (Gu et al., 2017; Zhu et al., 2018). In short, subordinates evaluate leadership styles such as tolerance and openness of inclusive leadership (Carmeli et al., 2010), different cognitive and affective responses will occur, and cognition-affection differences are likely to be the root cause of the double-edged sword effect of inclusive leadership. However, current research on the negative effects of inclusive leadership on subordinates’ behaviour outcomes mostly focuses on the innovation domain, such as creativity and innovative behaviour (Gu et al., 2017; Zhu et al., 2018), and the testing paths to explore both positive and negative effects of inclusive leadership are relatively single, focusing mainly on the cognitive level, such as psychological security and perception of dependence (Gu et al., 2017).

In view of this, this study will focus on both sides of the effect of inclusive leadership from two paths, cognition and affection, and select the typical and opposing manifestations of work behaviour of subordinates’ work withdrawal behaviour and proactive behaviour (Rego et al., 2018; Aggarwal et al., 2020) to discuss the following three questions. Firstly, what effect does inclusive leadership have on the two contrasting work behaviour of subordinates? Secondly, through what mediation mechanism is the effect transmitted? Thirdly, in what contexts does the mediation mechanism show differences in its effect?

In order to answer the above questions, this study provides an explanatory pathway based on Cognition-affection Personality System Theory (Zhao et al., 2021). It constructs a dual-path integration model concerning cognition-affection effect of inclusive leadership on subordinates’ work behaviour by using subordinate psychological entitlement and state gratitude as representative variables. These two variables were chosen because in relationship-oriented inclusive leadership situations, subordinates who perceive the leadership behaviour as their deserving of preferential treatment will develop psychological entitlement due to biassed subordinate cognitive evaluations (Dreiling, 2015), while subordinates who perceive the leadership behaviour as a favour from their superiors to their subordinates will develop gratitude (Harding et al., 2019).

Second, given the two-sided nature of the effect of inclusive leadership, this study further unpacked the indirect mechanisms of how inclusive leadership influences subordinate behaviour by using two prevalent and opposite in nature extra-role behaviour, proactive and work withdrawal behaviour. Furthermore, Cognition-affection Personality System Theory states cognition- affection response processes also vary from person to person and are influenced by individual characteristics (Mischel and Shoda, 1995). In today’s organisational context, where subordinates’ self-focus and affirmation tendencies are increasingly strong and prominent (Lin et al., 2022), this study introduces a subordinate personality characteristic variable called narcissistic personality within the context of Cognition-affection Personality System Theory, which is relevant to whether leadership behaviour can play an effective role and what role it plays (Zeigler-Hill et al., 2023), to examine its moderation role in the process of which inclusive leadership influences subordinates’ psychological entitlement and state gratitude.

**Figure 1:**
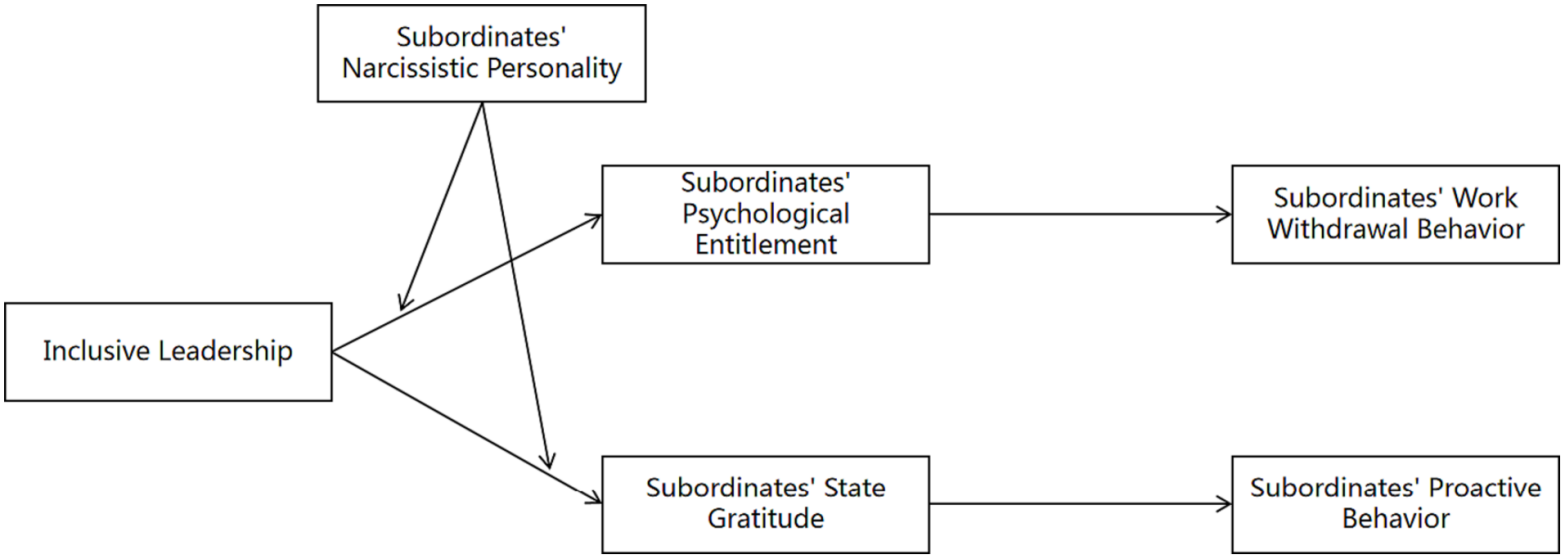
Theoretical model.

In summary, this study takes the double-edged sword effect of inclusive leadership as an entry point, incorporates the cognition-affection responses of subordinates to inclusive leadership into a logical framework, and analyses the influence mechanisms between inclusive leadership and subordinates’ work behaviour from two different mechanisms, with a view to providing theoretical explanations and empirical analyses for the existence of both positive and negative effects of inclusive leadership and the boundary conditions of the effects. At the same time, this study also helps organisational managers to be aware that inclusive leadership may bring both positive and negative results, and warns managers to demonstrate inclusive leadership behaviour in a reasonable manner so as to improve the effectiveness of inclusive leadership. The specific theoretical model is shown in Figure 1.

## Theoretical background and hypotheses development

### Cognition-affection personality system theory

Cognition-affection Personality System Theory suggests that external situational factors trigger internal cognitive or affective units that ultimately determine individuals’ attitudes and behaviour choices (Mischel and Shoda, 1995). At the same time, an individual’s internal cognitive and affective state has an information activation function (Rusting, 1998), which induces him or her to selectively identify and process information in the external environment in accordance with the principle of valence congruence (Yuan et al., 2019), which activates their cognitive or affective units through cognitive appraisal and ultimately determines his or her behaviour choices (Mischel and Shoda, 1995; Zhang et al., 2023).

The cognitive system is the individual’s informational control system, which uses external information to make rational and strategic behaviour. The affective system is the individual’s affective automatic system, which has the ability to respond to external stimuli in a manner similar to conditioned reflexes (Metcalfe and Mischel, 1999). Many scholars have confirmed that the process of influencing mechanisms in specific work events or leadership behaviour can provide good explanations for them based on Cognition-affection Personality Systems Theory (Gao and He, 2019; Zhao et al., 2021).

Therefore, leadership behaviour as an important situational factor influencing subordinates’ cognition and affection (Chen et al., 2022), can stimulate subordinates to pay attention to information in the work environment and process it under the mechanism of the cognition-affection personality system, thus forming cognitive evaluations and influencing subordinates’ cognition and emotion, and subsequently making corresponding behaviour decisions (Forgas and George, 2001). That is, inclusive leaders can provide signals to their subordinates, who may develop psychological entitlements (cognitive units) and state gratitude (affective units) through cognitive evaluations of the leaders’ behaviour (Zhang et al., 2023).

### Inclusive leadership, psychological entitlement and state gratitude

Inclusive leadership originated in the field of pedagogy and was introduced into leadership research by Nembhard and Edmondson (2006), who formally introduced the concept. As a typical relational leadership with which leaders are good at listening to the voices of subordinates, paying attention to their needs and focusing on interactions with them (Carmeli et al., 2010), inclusive leadership can effectively build harmonious organisational relationships and cope with the complexity and diversity of organisational management. From the perspective of characteristics, inclusive leadership has three dimensions of openness, effectiveness and accessibility (Carmeli et al., 2010). Although similar to styles such as servant leadership, spiritual leadership and ethical leadership (Nembhard and Edmondson, 2006; Brown and Mitchell, 2010; Carmeli et al., 2010), inclusive leaders are more attentive to the differentiated and diverse needs of their subordinates and expect to be able to stimulate their potential and energy (Hollander, 2009; Carmeli et al., 2010).

Psychological entitlement is a stable and pervasive subjective perception that individuals believe they deserve and are entitled to more preferential treatment and are exempt from social responsibility (Campbell et al., 2004; Gao et al., 2019). It has been shown that context-specific factors can stimulate individual psychological entitlement (Jordan et al., 2016; Qin et al., 2020). Therefore, based on Cognition-affection Personality System Theory, this study infers that inclusive leadership, as a situational variable, is likely to be an important antecedent for inducing the generation of psychological entitlement in subordinates.

On the one hand, inclusive leaders usually show an encouraging, forgiving and understanding attitude towards subordinates’ mistakes or errors in their work. The subordinate’s interpretation and processing of the leader’s behaviour, as well as his or her own experience of making mistakes without being reprimanded (Forgas and George, 2001), leads the subordinate to overestimate his or her status, competence and contribution to the organisation and to form a subjective perception that he or she deserves to be taken care of and treated favourably by the leaders (Dreiling, 2015).

On the other hand, inclusive leadership differs from other leadership styles (Qin et al., 2020) in that it focuses on “relationships” (Carmeli et al., 2010) and inclusive leaders are able to establish two-way, positive interactions with subordinates who easily interpret this high level of affective engagement as the leaders’ special behaviours to maintain a stable relationship between them. As a result, the subordinate processes this information to form a cognitive evaluation (Mischel and Shoda, 1995), which in turn activates the subordinate’s cognitive units and leads to psychological entitlements. Therefore, the following hypothesis is proposed.

*H*1: Inclusive leadership has a positive effect on psychological entitlement.

State gratitude is a positive emotional state that arises when individuals are favoured by others in certain events or situations (McCullough et al., 2001; Harding et al., 2019), which helps to enhance individual well-being (McCullough et al., 2002) and increases the frequency with which individuals engage in positive behaviours (Grant and Gino, 2010; Spence et al., 2014). Cognition-affection Personality System Theory suggests that leaders are the main interpersonal objects of subordinates in the organisation and their words and actions play an important role in influencing subordinates’ state gratitude (Ford et al., 2018; Wang and Chen, 2019).

Firstly, when inclusive leaders show a fault-tolerant attitude towards their subordinates’ faults at work, subordinates can feel the care and consideration from their leaders, which can enhance their own psychological security (Mansoor et al., 2020) and relieve stress at work (Ahmed et al., 2021). Secondly, inclusive leaders are willing to accept their subordinates’ different ways of working and give them the opportunity to try and make mistakes (Fang et al., 2019), so that they can feel trust, respect and recognition from their leaders (Tang et al., 2015). Finally, inclusive leaders are good at listening to their subordinates’ perspectives and actively provide guidance and assistance to their subordinates to the best of their ability (Carmeli et al., 2010), so their subordinates are fully engaged in their work with enthusiasm (Zeng et al., 2020).

Therefore, through the recognition and processing of these behaviour messages (Forgas and George, 2001), the subordinates are able to deeply recognise and feel the importance, support and high expectations of their leaders, thus contributing to the formation of their cognitive appraisal, which in turn activates the subordinates’ affective units and generates gratitude. In summary, the following hypothesis is proposed.

*H*2: Inclusive leadership has a positive effect on state gratitude.

The mediation role of psychological entitlement and state gratitude.

Work withdrawal is a deliberate negative behaviour response by employees to keep away from the organisation in the workplace (Gupta and Jenkins Jr, 1991) and is mainly manifested through tardiness, early leaving, slacking off during work hours, not doing their best and leaving work without a reason (Lehman and Simpson, 1992). Research has shown that individual perceptions are a significant factor in triggering subordinates’ work withdrawal behaviour (Wang and Yi, 2012), which can be tangibly or intangibly damaging to the individual himself as well as the organisation (Podsakoff et al., 2007; Ton and Huckman, 2008; Swider and Zimmerman, 2014).

Cognition-affection Personality System Theory suggests that an individual’s cognitive units are further motivated to engage in a specific behaviour when activated by situational factors (Mischel and Shoda, 1995). Therefore, this study holds that subordinates process the behaviour information of inclusive leaders and develop a strong perception of entitlement through cognitive evaluation (Tolmacz and Mikulincer, 2011). However, this psychological cognition further influences subordinates’ behaviour patterns and decisions (Zitek et al., 2010; Laird et al., 2015; Bai et al., 2017; Lee et al., 2019).

As a cognitive pathway, subordinates with higher psychological entitlement believe that they deserve more resources and care than others (Dreiling, 2015) and often show dissatisfaction with the current status quo, believing that they have the right to implement inappropriate work practices at work (Naseer et al., 2020). At the same time, subordinates with higher psychological entitlement tend to be self-centred, disregard organisational norms (Li, 2021), lack self-control (Raskin and Terry, 1988) and are more likely to engage in work withdrawal behaviour that are detrimental to organisational development. Therefore, the following hypothesis is proposed.

*H*3: Psychological entitlement mediates the relationship between inclusive leadership and work withdrawal behaviour.

Proactive behaviour is a spontaneous action taken by employees to make improvements to their job tasks or their own roles (Griffin et al., 2007) and is a voluntary, out-of-role behaviour that goes beyond what is required by job duties (Rego et al., 2018) and can help organisations to improve potential problems and increase overall organisational productivity (Kickul and Gundry, 2002; Meijerink et al., 2018; Wu et al., 2018). Combined with the perspective of Cognition-affection Personality System Theory, as an affective pathway, subordinate state gratitude then triggers positive behaviour corresponding to it (Michie, 2009; Tian et al., 2016; Ford et al., 2018).

That is, during interactions with inclusive leaders, subordinates develop gratitude towards the leaders through cognitive appraisals. Subordinates with state gratitude usually hold positive and optimistic attitudes at work (Fehr et al., 2017), they are able to integrate and process organisational information autonomously, find ways and means to resolve internal problems, and are willing to invest a lot of their own time and energy in extra-role behaviour that reflect their own values (Parker et al., 2010). At the same time, subordinates often attribute their state of gratitude to receiving “care” from their leaders, which inspires a sense of responsibility to give back to the leaders and the organisation (Bonnie and Waal, 2004), leading them to put more efforts and awareness into completing their current work and to actively engage in proactive behaviour that benefit the organisation and other people. Therefore, the following hypothesis is proposed.

*H*4: State gratitude mediates the relationship between inclusive leadership and subordinates' proactive behaviour.

### The moderation role of narcissistic personality

The study of subordinates’ cognitive and affective reactions to leadership in Chinese organisational contexts cannot be separated from the influencing factors of personality traits. Narcissistic personality, a typical dark personality trait, is characterise by excessive arrogance and self-love, a strong sense of psychological superiority and an inflated view of the self (Lin et al., 2022). According to Cognition-affection Personality System Theory, individual traits can provide an explanation for the relationship between external situations and their cognitive and affective responses (Mischel and Shoda, 1995), That is., a subordinate’s narcissistic personality affects his or her level of understanding of leadership behaviour (Zeigler-Hill et al., 2023).

Specifically, subordinates with higher narcissistic personalities typically overestimate and exaggerate themselves (Dreiling, 2015), believe themselves to be superior (O’Reilly and Pfeffer, 2021), and ignore the interests of the organisation and other people (Lin et al., 2022). Because subordinates with high narcissistic personalities have high levels of self-confidence in themselves as well as self-superiority (Krizan and Herlache, 2018), subordinates are more likely to form inflated self-evaluation through interpretation faced with the leader’s fault tolerance, respect, and support (Lin et al., 2022), which are the source of their psychological entitlement (Harvey and Dasborough, 2015). Furthermore, subordinates with higher narcissistic personalities are self-centred, greedy and lack empathy (Williams and Williams, 2017) and they consider themselves to have a certain degree of privilege (Fehn and Schütz, 2021), resulting in a lower level of gratitude towards inclusive leadership (Zeigler-Hill et al., 2023). As a result, subordinates’ levels of state gratitude towards inclusive leaders may subsequently decrease. Therefore, the following hypotheses are proposed.

*H*5: Narcissistic personality plays a moderation role between inclusive leadership and psychological entitlement. That is, the higher the narcissistic personality is, the stronger the positive relationship between inclusive leadership and psychological entitlement is.

*H*6: Narcissistic personality plays a moderation role between inclusive leadership and state gratitude. That is, the higher the narcissistic personality is, the weaker the positive relationship between inclusive leadership and state gratitude is.

#### Moderated mediation role

Combining the above hypotheses, this study further infers that the mediation effects of subordinates’ psychological entitlement and state gratitude may be influenced by their narcissistic personality. According to Cognition-affection Personality System Theory, it is known that individual traits, cognition and emotion interact in the behaviour choices of individuals (Mischel and Shoda, 1995). That is, for subordinates with high narcissistic personalities, their inflated self-perceptions, high confidence in competence, and self-affirming tendencies (O’Boyle et al., 2012; O’Reilly and Pfeffer, 2021), perceive the leadership behaviour of inclusive leaders as a form of privilege and preferential treatment that they deserve and thus increase their perception of psychological entitlement, inducing more work withdrawal behaviour in their subordinates. Conversely, egoistic, self-focused, and empathy deficiency traits (Hepper et al., 2014) reduce or block the effect of inclusive leadership on subordinates’ gratitude, which in turn can reduce the implementation of proactive behaviour at work. Therefore, the following hypotheses are proposed.

*H*7: Narcissistic personality moderates the mediation role of psychological entitlement between inclusive leadership and subordinate work withdrawal. That is, the higher the narcissistic personality is, the stronger the mediation role of psychological entitlement between inclusive leadership and subordinate work withdrawal behaviour is.

*H*8: Narcissistic personality moderates the mediation role of state gratitude between inclusive leadership and subordinates' proactive behaviour. That is, the higher the narcissistic personality is, the weaker the mediation role of state gratitude between inclusive leadership and subordinates' proactive behaviour is.

## Methods

### Sample and procedure

The data source for this study was a sample of employees and their direct leaders from five service companies engaged in banking, security, escorting, security inspection and security technology prevention in China, and the data was collected using offline questionnaires. To reduce the impact of homology bias, this study conducted a 1:1 employee-direct leader pairing at three time points, with a time interval of 1 month. The specific survey process was as follows: at time point 1 (T1) the survey was conducted with the employees including basic information about the employee and inclusive leadership; at time point 2 (T2) the survey was conducted with the employees including psychological entitlement, state gratitude and narcissistic personality; at time point 3 (T3) the survey was conducted with the employees’ direct leaders including the employee’s work withdrawal behaviour and proactive behaviour. With the exception of some demographic variables, all questionnaires in this study were scored on a 6-point Likert scale.

In order to enable participants to complete the questionnaire correctly and effectively, we took the following four steps. Firstly, before the questionnaires were distributed, we explained to all participants that the data from the questionnaires was collected for academic research purposes only and not for any other purposes. Secondly, we gave each person a gift (worth approximately 10 yuan RMB) after completing the survey each time. Thirdly, one of our members maintained a close relationship with the participants during the process of completing the questionnaire to address any questions they had. Finally, after participants completed the questionnaires, we checked them and collected, sealed and coded them immediately.

In the first survey, 563 staff questionnaires were distributed on site and a total of 544 valid questionnaires were returned; in the second survey, 535 valid questionnaires were distributed to employees who provided valid questionnaires in the first survey; in the third survey, 518 questionnaires were distributed to the leaders of employees who provided valid questionnaires in the second survey, resulting in 518 valid matching questionnaires between employees and leaders, with a return rate of 92.01%. In terms of the structure of the sample, the majority of employees were male, accounting for 50.2% of the total sample; in terms of age structure, the majority of employees were young people, with 82% of employees under the age of 35; in terms of education structure, 83% of the total sample size were of or above undergraduate. The basic information of the samples is shown in Table 1.

**Table 1:** Basic information of samples.

Variable	Attribute	Number	Percentage
Age	≤25	56	10.8
	26–30	113	21.8
	31–35	256	49.4
	36–40	47	9.1
	41–45	24	4.6
	46–50	17	3.3
	≥51	5	1
Sex	Male	260	50.2
	Female	258	49.8
Education level	Senior high	32	6.2
	Junior college	109	21
	Undergraduate	289	55.8
	Master	77	14.9
	Doctor	11	2.1

*N* = 518.

### Measurements

The scales used in this study are mature ones used by many scholars at home and abroad, and each question item was scored on a 6-point Likert scale, measuring the six main variables of inclusive leadership, state gratitude, psychological entitlement, proactive behaviour, work withdrawal behaviour and narcissistic personality.

For inclusive leadership (T1): The Inclusive Leadership Scale developed by Carmeli et al. (2010) was used, with nine questions. The representative question is “My leader is open to new ideas” and the scale has a Cronbach’s alpha value of 0.93.

For psychological entitlement (T2): The Psychological Entitlement Scale developed by Yam et al. (2017) was used, with four questions. The representative question is “I genuinely feel that I should enjoy more rights than other colleagues.” The Cronbach’s alpha value for this scale is 0.89.

For state gratitude (T2): The State Gratitude Scale developed by Spence et al. (2014) was used, with five questions. The representative question is “I feel happy because of my leader’ help at work” and the scale has a Cronbach’s alpha value of 0.87.

For work withdrawal (T3): The Work Withdrawal Scale developed by Lehman and Simpson (1992) was used, with 12 questions. The representative question is “The employee will be distracted at work” and the scale has a Cronbach’s alpha value of 0.86.

For proactive behaviour (T3): The Proactive Behaviour Scale developed by Frese and Fay (2001) was used, with seven questions. The representative question is “The employee will deal with problems in a positive way” and the scale has a Cronbach’s alpha value of 0.87.

For narcissistic personality (T2): The Narcissistic Personality Scale developed by Jones and Paulhus (2014) was used, with nine questions. The representative question is “I think I’m great because everyone says so to me” and the scale has a Cronbach’s alpha value of 0.92.

For the measurement of control variables, with reference to previous studies (Chen et al., 2022; Wei et al., 2023), this study selected employees’ age, gender, and education level from common demographic variables as control variables. Among them, the age of employees was divided into 7 ranges including 25 years old and below, 26–30 years old, 31–35 years old, 36–40 years old, 41–45 years old, 46–50 years old, 51 years old and above which is approximate a continuous scale. Gender was dummy coded, 0 for male and 2 for female. The education level of employees was divided into senior high school, junior college, undergraduate, master’s, doctoral, which was also treated as a continuous scale.

### Statistical analyses and results

#### Confirmatory factor analysis

In this study, Mplus 7.4 was used to perform confirmatory factor analysis on related variables to test the discrimination validity between variables. The results are shown in Table 2, the six factor model has the best fitting effect (χ^2^/= 335.22, *df* = 155, χ^2^/*df* = 2.16, CFI = 0.96, TLI = 0.95, RMSEA = 0.05, SRMR = 0.05), indicating that the six variables in this study have good discriminant validity.

**Table 2:** Confirmatory factor analysis (CFA) results of measurement models.

Models	Description	*χ*^2^	*df*	*χ*^2^/*df*	CFI	TLI	RMSEA	SRMR
Model a	One-factor model	3181.11	170	18.71	0.28	0.20	0.21	0.21
Model b	Two-factor model	1938.43	169	11.47	0.58	0.53	0.16	0.15
Model c	Three-factor model	1250.23	167	7.49	0.74	0.71	0.13	0.13
Model d	Four-factor model	955.08	164	5.82	0.81	0.78	0.11	0.12
Model e	Five factor model	678.96	160	4.24	0.88	0.85	0.09	0.10
Model f	Six-factor model	335.22	155	2.16	0.96	0.95	0.05	0.05

*N* = 518; a all six variables combined as the same factor; b inclusive leadership, psychological entitlement, state gratitude, proactive behaviour and work withdrawal behaviour were combined as the same factor; c psychological entitlement, state gratitude, proactive behaviour and work withdrawal behaviour were combined as the same factor; d state gratitude, proactive behaviour and work withdrawal behaviour were combined as the same factor; f hypothetical model.

Due to the fact that the four variables of psychological entitlement, state gratitude, narcissistic personality, and inclusive leadership have the same source, in order to avoid the influence of common method bias on the research results, this study used Harman’s singleton test to test for common method bias. All variables were placed in an exploratory factor analysis to test the results of non-rotated factor analysis. The results indicate that the variance explained by the first factor is 27.21%, which is less than the critical standard of 40%. In addition, this study also used the Unmeasured Latent Method Construct for controlling unmeasured potential method bias to test for common method bias. After incorporating method factors into the model, the fitting indicators of the model are: χ^2^/*df* = 1.79, CFI = 0.97, TLI = 0.96, RMSEA = 0.04, SRMR = 0.05. Compared with the model before control, the improvement in CFI, TLI, and RMSEA of the model after adding method factors is less than 0.02, indicating that the fitting degree of the model has not been significantly improved. In summary, there is no serious common method bias in this study.

### Correlation analysis

The means, standard deviations and correlation coefficient matrices of the variables in this study are shown in Table 3. It can be seen that inclusive leadership is significantly and positively related to psychological entitlement (*γ* = 0.28, *p* < 0.01) and state gratitude (*γ* = 0.11, *p* < 0.01); psychological entitlement is significantly and positively related to work withdrawal behaviour (*γ* = 0.32, *p* < 0.01); state gratitude is significantly and positively related to proactive behaviour (*γ* = 0.20, *p* < 0.01), and the results of the above analyses tentatively support the related hypotheses in this study.

**Table 3:** Means, standard deviations and correlation coefficients among main variables.

	Mean (*M*)	Standard deviation (*SD*)	1	2	3	4	5	6	7	8	9
1.Age	29.96	6.92									
2.Gender	0.50	0.50	−0.04								
3.Education level	4.20	1.02	−0.07	0.01							
4.Inclusive leadership	5.06	0.93	−0.04	0.10*	0.01	(0.93)					
5.Psychological entitlement	5.15	0.69	−0.07	0.02	0.04	0.28**	(0.89)				
6.State gratitude	5.23	0.64	0.02	−0.02	−0.10*	0.11**	0.03	(0.87)			
7.Work withdrawal behaviour	5.00	0.78	−0.02	0.02	0.03	0.38**	0.32**	−0.07	(0.86)		
8.Proactive behaviour	5.30	0.57	−0.12**	−0.07	0.03	0.12**	−0.05	0.20**	−0.06	(0.87)	
9.Narcissistic personality	2.59	1.22	0.06	−0.05	0.06	−0.12**	0.003	−0.35**	−0.38**	−0.21**	(0.92)

*N* = 518; **p < 0.01, *p < 0.05 respectively.

### Test of main effects

**Figure 2:**
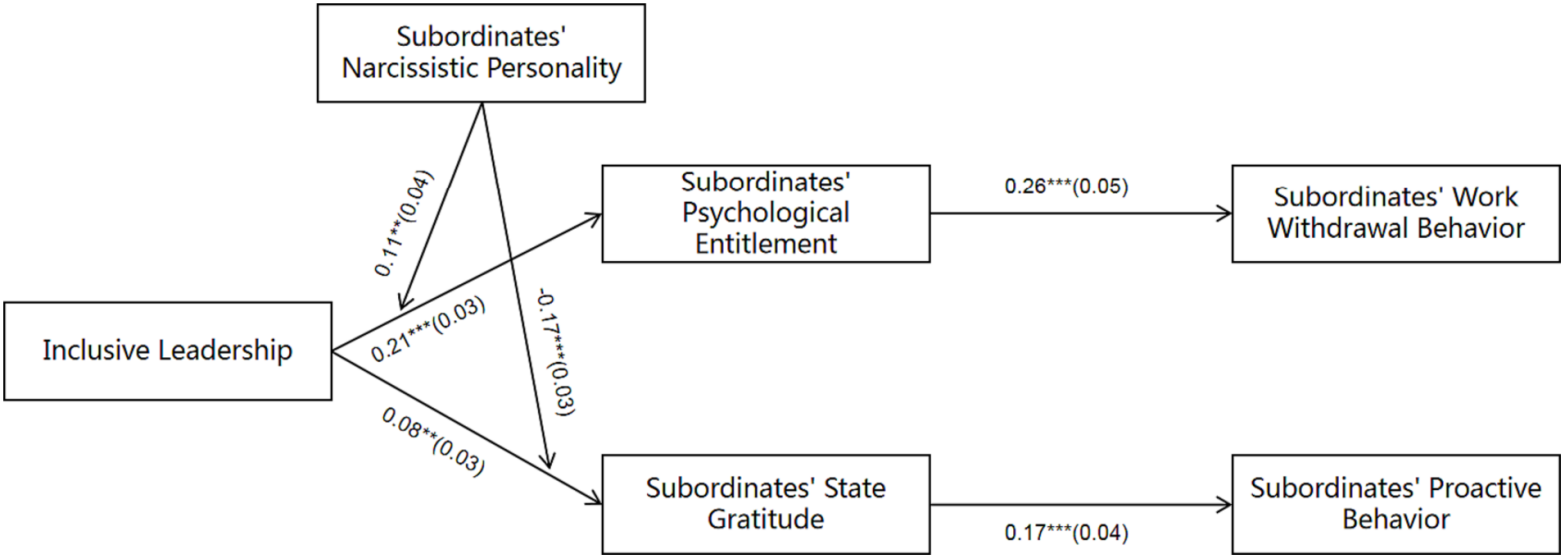
Path coefficients. ***, **, * stands for *p* < 0.001, *p* < 0.01, *p* < 0.05 respectively; coefficients in the graph are standardised coefficients with standard errors in parentheses; control variables are age, gender and education.

Mplus 7.4 was used to test the fitting indexes and related hypotheses of structural equation model. Firstly, according to the fitting indexes of the theoretical model (*x^2^* = 405.63, *df* = 159, *x^2^/df* = 2.55, CFI = 0.94, TLI = 0.93, RMSEA = 0.06, SRMR = 0.07), it can be seen that the fitting of the model is good. Secondly, the results of the path analysis are shown in Figure 2. Inclusive leadership has a positive effect on psychological entitlement (*β* = 0.21, *p* < 0.001) and state gratitude (*β* = 0.08, *p* < 0.01), so H1 and H2 are verified.

### Test of mediation effects

In this study, Bootstrap (repeated sampling 5,000 times) was used to test the mediation effects of psychological entitlement and state gratitude respectively, and the results are shown in Table 4. The mediation effect of psychological entitlement (*β* = 0.05, *p* < 0.01) was significant and the 95% confidence interval was [0.026, 0.087], which did not contain 0; the mediation effect of state gratitude (*β* = 0.01, *p* < 0.05) was significant and the 95% confidence interval was [0.003, 0.025], which did not contain 0. Therefore, H3 and H4 are verified.

**Table 4:** Results of mediation effects of psychological entitlement and state gratitude.

Indirect path	Indirect effect *β*	95%confidence interval
Path 1: Inclusive leadership → psychological entitlement → work withdrawal behaviour	0.05**	[0.026, 0.087]
Path 2: Inclusive leadership → state gratitude → proactive behaviour	0.01*	[0.003, 0.025]

*N* = 518; Bootstrap = 5,000.

### Test of moderation effects

**Figure 3:**
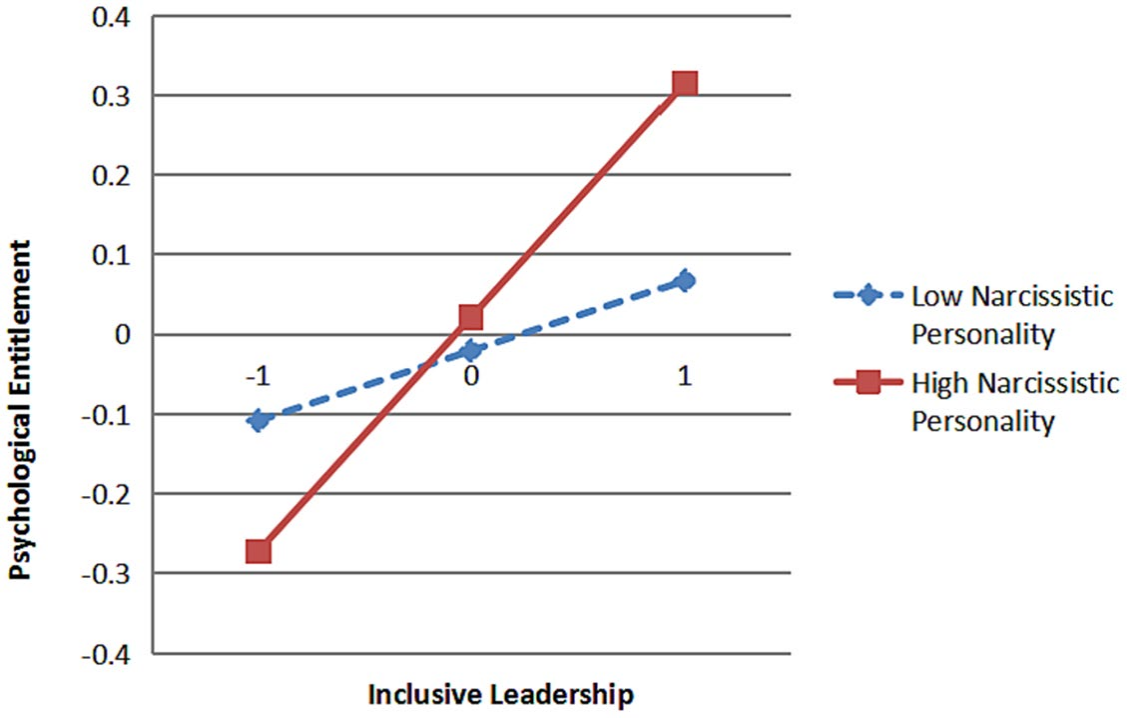
The moderation effect of narcissistic personality between inclusive leadership and psychological entitlement.

**Figure 4:**
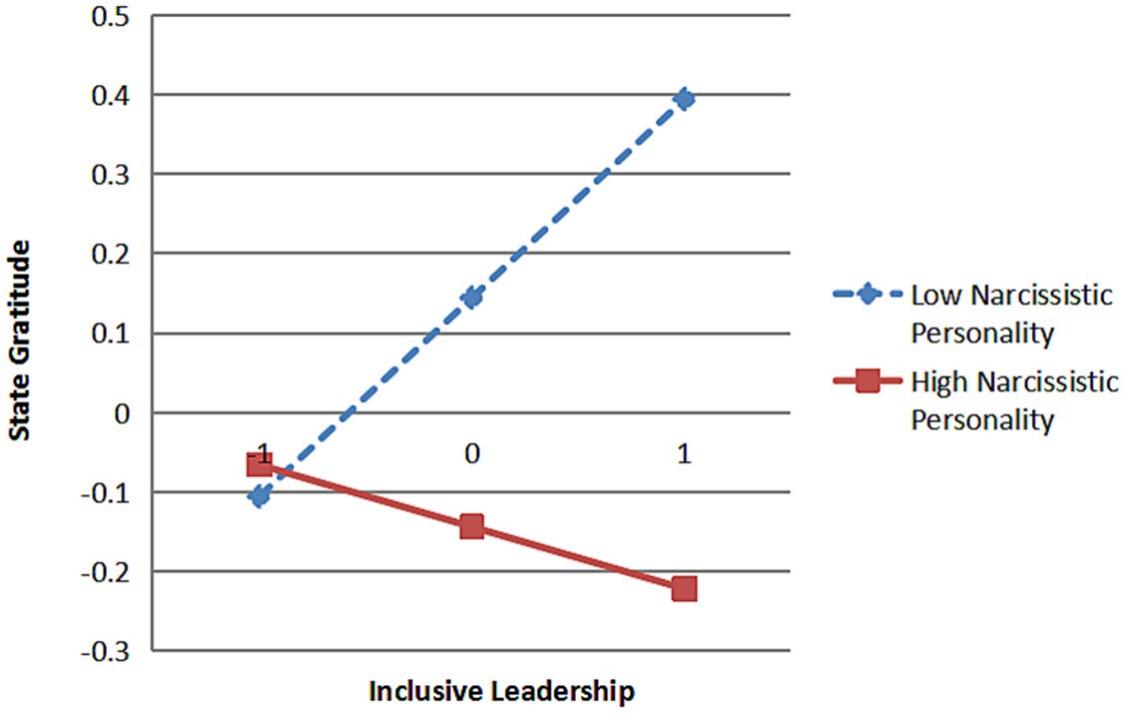
The moderation effect of narcissistic personality between inclusive leadership and state gratitude.

From Figure 2, it can be concluded that the interaction between inclusive leadership and narcissistic personality has a significant path effect on psychological entitlement (*β* = 0.11, *p* < 0.01) and state gratitude (*β* = −0.17, *p* < 0.001), indicating that narcissistic personality significantly moderates the relationship between inclusive leadership and psychological entitlement as well as the relationship between inclusive leadership and state gratitude. To further explain the moderation effect relationship of narcissistic personality, a simple slope test was conducted as suggested by Aiken and West (1991) and plotted as shown in Figures 3, 4.

The results indicate that when subordinate narcissistic personality is low, inclusive leadership has a non-significant positive effect on psychological entitlement (*β* = 0.06, *t* = 1.01, *p* = 0.314), however, inclusive leadership has a stronger positive effect on state gratitude (*β* = 0.29, *t* = 5.60, *p* < 0.001); when subordinate narcissistic personality is high, the positive effect of inclusive leadership on psychological entitlement is significantly enhanced (*β* = 0.32, *t* = 6.56, *p* < 0.001) while the positive effect of inclusive leadership on state gratitude is significantly weakened (*β* = −0.12, *t* = −2.82, *p* < 0.01). That is, the higher the narcissistic personality of subordinates is, the stronger the positive effect of inclusive leadership on their psychological entitlement is, while the positive effect of inclusive leadership on state gratitude is greatly weakened, so H5 and H6 are verified.

To test the moderation effect of narcissistic personality, this study used Bootstrap (repeated sampling 5,000 times) to test the moderated mediation effect. The results are shown in Table 5, the mediation effect of psychological entitlement between inclusive leadership and work withdrawal behaviour is moderated by narcissistic personality. That is, for subordinates with higher narcissistic personality (one standard deviation above the mean), the indirect effect of inclusive leadership through psychological entitlement on work withdrawal behaviour is significantly higher than subordinates with lower narcissistic personality (one standard deviation below the mean), the difference is significant (*β* = 0.07, *p* < 0.05) and the 95% confidence interval is [0.018, 0.129], excluding 0. Therefore, H7 is verified.

**Table 5:** Test results of moderated mediation effects.

Narcissistic personality	Inclusive leadership → psychological entitlement → work withdrawal behaviour		Inclusive leadership → state gratitude → proactive behaviour	
	Indirect effect *β*	95% confidence interval CI	Indirect effect *β*	95% confidence interval CI
Low narcissistic personality	0.02	[−0.015, 0.062]	0.04**	[0.020, 0.073]
High narcissistic personality	0.08***	[0.043, 0.129]	−0.02*	[−0.033, −0.006]
Differences	0.07*	[0.018, 0.129]	−0.06**	[−0.100, −0.029]

*N* = 518; Bootstrap = 5,000.

The mediation effect of state gratitude between inclusive leadership and proactive behaviour is moderated by narcissistic personality. That is, for subordinates with higher narcissistic personality (one standard deviation above the mean), the indirect effect of inclusive leadership on proactive behaviour through state gratitude is significantly lower than subordinates with lower narcissistic personality(one standard deviation below the mean), the difference is significant (*β* = −0.06, *p* < 0.01) and the 95% confidence interval is [−0.100, −0.029], excluding 0. Therefore, H8 is verified.

## Discussion

### Theoretical contributions

Firstly, studies on the effect of inclusive leadership on subordinates’ work behaviour are mostly positive (Javed et al., 2019; Qi et al., 2019), although a few scholars have also mentioned the potential negative effects of inclusive leadership, but they mostly focus on creativity and innovative behaviour (Gu et al., 2017; Zhu et al., 2018), which is not conducive to academics’ systematic and complete understanding of the role of inclusive leaders on their subordinates. This study takes an alternative approach by choosing two opposing extra-role behaviour, namely work withdrawal behaviour and proactive behaviour, to confirm the double-edged sword effect of inclusive leadership and explain the internal mechanism of the relationship between inclusive leadership and subordinates’ work behaviour, which to a certain extent complements and broadens the scope of inclusive leadership research and helps scholars to understand, focus on and reflect the impact of inclusive leadership in organisations from different research perspectives in a more profound and comprehensive manner from different research perspectives.

Secondly, the mechanisms of inclusive leadership’s effects on subordinates’ cognition and emotion in previous studies have mostly been explored from a positive perspective (Ye et al., 2017; Wang et al., 2019). This study takes a dialectical perspective and adopts Cognition-affection Personality System Theory as a theoretical framework to construct a holistic logic among the research variables and a complete action chain of “leadership style→subordinate cognition/emotion→subordinate behaviour” to further understand the leadership effectiveness of inclusive leaders in a comprehensive manner. This study examines subordinates’ perceptions, interprets the psychological entitlement triggered by inclusive leadership behaviour, and stimulates subordinates’ state gratitude. Through theoretical and empirical research, it provides a more complete explanation of the effect mechanism between inclusive leadership and subordinate behaviour, offering a new theoretical perspective on the study of the effect mechanism of inclusive leadership.

Finally, this study includes subordinate narcissistic personality in the research framework to examine its moderation effect on the dual cognitive and affective pathways of inclusive leadership in affecting subordinates. Currently, research on narcissistic personality is more often explored as an independent variable (Zagenczyk et al., 2017; Treadway et al., 2019), and this study verifies that the direct effect of inclusive leadership on subordinates’ psychological entitlement, state gratitude, and the indirect effect of subordinates’ work withdrawal and proactive behaviour can vary depending on the level of subordinates’ narcissistic personality. This study also verifies that the direct effects of inclusive leadership on subordinates’ psychological entitlement, state gratitude and indirect effects on subordinates’ work withdrawal behaviour and proactive behaviour vary depending on the level of subordinates’ narcissistic personality. This study contributes to further deepening the academic understanding and development of subordinate narcissistic personality, as well as providing a useful supplement to the research on the moderation mechanisms of inclusive leadership affecting effects.

### Practical implications

Firstly, organisations should regard inclusive leadership in a dialectical way and try to curb its negative effects as much as possible. While promoting inclusive leadership behaviour, organisations should also be aware that leaders who are always inclusive do not necessarily produce good results. Therefore, organisations need to prevent and warn against the negative effects of inclusive leadership. Specifically, leaders should maintain a moderate level of inclusive leadership behaviour towards their subordinates and give them appropriate criticism, rather than just being “yes-man,” so as not to induce more serious mistakes by subordinates due to excessive “fault tolerance” by leaders. At the same time, organisations should establish an effective feedback mechanism to achieve two-way communication between leaders and subordinates, and pay attention to behaviour boundaries when cultivating relationships with subordinates, so as to avoid the negative effects of inclusive leadership behaviour. In addition, organisations can also strengthen their leaders’ behavioural skills through targeted leadership training (Zhu et al., 2018).

Secondly, organisations should pay attention to the psychological changes of subordinates and create a culture of gratitude. On the one hand, leaders should intervene in the psychological entitlement levels of their subordinates, actively guide them to make accurate self-evaluations and reduce their excessive expectations of privilege at work. At the same time, leaders should grasp the psychological state of their subordinates through psychological training and assessment in order to achieve the optimal effect of human resources management. On the other hand, gratitude is a two-way behaviour, and leaders should advocate a grateful organisational culture atmosphere, actively establish an image of gratitude, appropriately carry out group activities about gratitude, properly guide subordinates to express gratitude, internalise frequent acts of kindness, consciously cultivate a grateful mindset among subordinates, and form good relationships with them in a grateful atmosphere (Algoe et al., 2013).

Thirdly, organisations should pay attention to the personality differences of subordinates and make the best use of their talents. On the one hand, organisations should actively use third-party evaluations when recruiting employees. Employees with high narcissistic personalities tend to be more confident in interviews, and they are more likely to be favoured by interviewers, but their blind arrogance in the workplace can pose a serious threat to the organisations. Organisations can therefore set up probationary assessments to see if employees with narcissistic personalities can be better integrated into the organisations. For employees on service now, managers should identify their values and strengths in the process of getting along at work, so that employees can play an effective role in their areas of expertise (Zeigler-Hill et al., 2023). On the other hand, organisations should provide appropriate psychological counselling and guidance to help subordinates recolonize and face up to themselves, and encourage them to participate in relevant training to curb the spread of the negative effects of narcissistic personality.

### Limitations and future research

There are still some limitations and shortcomings in this study that need to be further explored in the future research. Firstly, this study has confirmed the double-edged sword effect of inclusive leadership from a cognition-affection perspective. In the future, other research perspectives (e.g., Dominance Compensation Theory, Personal-environmental Matching Theory) can be introduced or other variables that have not yet been focused on can be further explored and the differences in their mechanisms of action can be compared based on our existing findings.

Secondly, this study explores the mechanisms of inclusive leadership, subordinates’ work withdrawal behaviour and proactive behaviour mainly at the individual level, and further research on the double-edged sword effect of inclusive leadership on teams or organisations could be explored by attempting to measure team-level variables.

Third, the control variables included in this study are limited, and other control variables that may affect the findings, such as time spent with leaders, leader member exchange, and compensation incentives, can be added to the study design in the future.

Fourth, this study only focuses on the moderation effect of narcissistic personality, and future research could consider other potential moderation variables (e.g., competitive climate, transnational thinking, etc.) in order to explore deeper into the boundary conditions between inclusive leadership and subordinates’ work behaviour.

Fifth, the cross-sectional data used in this study may not accurately reveal the dynamic processes between the variables, and future research could be conducted through experimental research methods or log tracking to make the findings more precise and credible.

Sixth, due to our limited social resources, the sample of this study mainly focuses on data findings from a local area in China, and the external validity of the sample categories may have certain shortcomings, and the generality of the findings needs to be further confirmed. In the future, the sample scope can be expanded or cross-cultural studies can be conducted to obtain samples from different countries, regions, enterprises and populations to make the results more convincing.

## Conclusion

Based on the Chinese cultural context, this study explores the double-edged sword effect of inclusive leadership from a dialectical perspective. Bases on Cognition-affection Personality System Theory, this study constructs a cognitive and emotional dual-path integration model of inclusive leadership affecting subordinates’ work behaviour, and further explores the boundary conditions of inclusive leadership mechanisms by introducing subordinates’ narcissistic personality as a moderation variable, providing a new direction for inclusive leadership research.

This study collected data through a three-stage paired questionnaire, and the results are showed as follows. Inclusive leadership leads to psychological entitlement in subordinates, which in turn leads to work withdrawal behaviour. Inclusive leadership also triggers state gratitude in subordinates, which in turn motivates proactive behaviour. In addition, the overall indirect effect of the dual path of inclusive leadership on subordinates’ withdrawal and proactive behaviour is positive. That is, inclusive leadership indirectly contributes positively to subordinates’ withdrawal behaviour through their psychological entitlement and to their proactive behaviour through state gratitude.

Further, subordinate narcissistic personality not only positively moderates the relationship between inclusive leadership and subordinate psychological entitlement, but also significantly moderates the indirect effect of psychological entitlement on the relationship between inclusive leadership and work withdrawal behaviour. That is, the higher the subordinate narcissistic personality is, the stronger the indirect effect is, and the weaker the opposite. Subordinate narcissistic personality not only negatively moderates the relationship between inclusive leadership and subordinate state gratitude, but also significantly moderates the indirect effect of state gratitude on the relationship between inclusive leadership and proactive behaviour. That is, the higher the subordinate narcissistic personality, the weaker the indirect effect is, and the stronger the opposite. The above findings have implications for both practical management and theoretical development in organisations.

## Data availability statement

The original contributions presented in the study are included in the article/supplementary material, further inquiries can be directed to the corresponding author.

## Ethics statement

Ethical review and approval was not required for the study on human participants in accordance with the local legislation and institutional requirements. Written informed consent from the patients/ participants OR patients/participants legal guardian/next of kin was not required to participate in this study in accordance with the national legislation and the institutional requirements.

## Author contributions

HC: Data curation, Investigation, Project administration, Visualization, Writing – original draft, Writing – review & editing. LW and JB: Investigation, Resources, Writing – review & editing. JB and ZZ: Formal analysis, Methodology, Writing – review & editing.
